# Association of Carbapenemase-Producing *Enterobacterales* Detected in Stream and Clinical Samples

**DOI:** 10.3389/fmicb.2022.923979

**Published:** 2022-06-09

**Authors:** Gyung-Hye Sung, Si Hyun Kim, Eun Hee Park, Suk Nam Hwang, Jea-Dong Kim, Gyu Ri Kim, Eun-Young Kim, Joseph Jeong, Sunjoo Kim, Jeong Hwan Shin

**Affiliations:** ^1^Busan Institute of Health and Environment, Busan, South Korea; ^2^Department of Clinical Laboratory Science, Semyung University, Jecheon, South Korea; ^3^Ulsan Health and Environment Research Institute, Ulsan, South Korea; ^4^Gyeongsangnam-do Provincial Government Health and Environment Institute, Jinju, South Korea; ^5^Department of Laboratory Medicine and Paik Institute for Clinical Research, Inje University College of Medicine, Busan, South Korea; ^6^Department of Laboratory Medicine, University of Ulsan College of Medicine, Ulsan University Hospital, Ulsan, South Korea; ^7^Department of Laboratory Medicine, Gyeongsang National University College of Medicine, Jinju, South Korea

**Keywords:** carbapenem, carbapenemase-producing *Enterobacterales*, antimicrobial resistance, stream, multi-locus sequence typing

## Abstract

**Background:**

The spread of carbapenem-resistant *Enterobacterales* (CRE) strains has caused treatment failure and is a worldwide threat to public health. However, there are limited reports on the prevalence of carbapenemase-producing *Enterobacterales* (CPE) in aquatic environments and its association with clinical isolates. This study aimed to investigate the prevalence of CPE in a stream environment and its genetic relationship with clinical isolates in Korea.

**Methods:**

A total of 4,582 water samples were collected from 94 streams. Multiplex PCR and sequencing were used to detect and identify six carbapenemase genes. Multi-locus sequence typing (MLST) was performed to investigate the genetic relatedness between the environmental strains and clinical isolates.

**Results:**

A total of 133 CRE strains were isolated from the streams. *Klebsiella pneumoniae* was the most common CRE (45.9%), followed by *Enterobacter cloacae* complex (29.3%), *Escherichia coli* (13.5%), *Raoultella ornithinolytica* (5.3%), and *Citrobacter freundii* (2.3%). Ninety (67.7%) isolates carried carbapenemase genes. *K. pneumoniae* carbapenemase-2 (36.7%) and New Delhi metallo-β-lactamase-5 (32.2%) were the common carbapenemases detected. Sequence type (ST)307 and ST11 *K. pneumoniae* strains harboring the *bla*_KPC-2_ gene were the most prevalent in stream and patient samples.

**Conclusion:**

CPE was highly prevalent in streams and closely related to the isolates obtained from patients. Therefore, continuous monitoring of stream environments is required to control the spread of carbapenem resistance.

## Introduction

Carbapenems, such as imipenem, meropenem, doripenem, and ertapenem, are the last choice for the treatment of gram-negative bacteria (Pitout, 2010). However, the spread of carbapenem-resistant strains has caused treatment failure and is a worldwide threat to public health (Nordmann et al., 2011). Carbapenem-resistant *Enterobacterales* (CRE) were uncommon before 2000 but have been prevalent worldwide since the emergence of *Klebsiella pneumoniae* carbapenemase (KPC)-producing strains (Yigit et al., 2001).

Recently, the “One Health” concept was introduced to control antimicrobial resistance (Robinson et al., 2016). This concept highlights the interconnected nature of human, animal, and environmental health. The environment is a hotspot for the development and spread of antimicrobial resistance genes (Martinez, 2009). Several studies have focused on the role of aquatic environments contaminated with livestock or human waste, or hospital wastewater (Reinthaler et al., 2003; Diwan et al., 2010). The isolation of carbapenemase-producing *Enterobacterales* (CPE) in rivers has been reported in a few studies (Aubron et al., 2005; Piedra-Carrasco et al., 2017); however, reports revealing the prevalence of CPE in aquatic environments and their association with clinical isolates are limited.

This study aimed to investigate the prevalence of CPE in a stream environment and its genetic relationship with clinical isolates in Korea.

## Materials and Methods

### Collection and Identification of Strains

We selected 94 streams in both urban areas and outside the city, flowing from Busan, Ulsan, and Gyeongsangnamdo in Korea. A total of 4,582 water samples were collected from 218 sites in 94 streams between July 2017 and August 2019.

The water samples were filtered through sterile (0.45 μm) membrane filters (Merck Millipore, Billerica, MA, United States). The surface of the filtered membrane was collected using sterile cotton swabs and cultured on CHROMagar™ KCP plates (CHROMagar Microbiology, Paris, France) at 35°C for 48 h. Suspected CRE colonies grown on the medium were sub-cultured on blood agar plates at 35°C for 24 h. Colonies were identified using VITEK MS (BioMerieux, Marcy I’Etoile, France).

CPE isolates from humans were included to investigate the association between environmental and clinical strains. We selected clinical strains by matching environmental strains with species, CPE genes, periods, and regions (Kim et al., 2020).

### Antimicrobial Susceptibility

The minimum inhibitory concentrations of meropenem, imipenem, and ertapenem were measured using the E test (BioMerieux, Marcy I’Etoile, France). An additional antimicrobial susceptibility test was performed using the disk diffusion method for all CRE isolates. The following antimicrobial agents were used: ampicillin, piperacillin, ampicillin–sulbactam, cefazolin, cefotaxime, ceftazidime, cefepime, aztreonam, cefoxitin, amikacin, gentamicin, ciprofloxacin, trimethoprim/sulfamethoxazole, tigecycline, and amoxicillin/clavulanic acid. The results were interpreted according to the guidelines of the Clinical and Laboratory Standard Institute (CLSI M100 30th ed., 2020).

### Molecular Characterization and Multi-Locus Sequence Typing

Multiplex PCR and sequencing were performed to detect and identify six carbapenemase genes (*bla*_KPC_, *bla*_VIM_, *bla*_NDM,_ *bla*_IMP_, *bla*_OXA_, and *bla*_GES_), as described previously (Lee et al., 2018; Kim et al., 2019). MLST determined the genetic relatedness of the isolates. MLST was performed on 240 CPE strains, including *K. pneumoniae, Escherichia coli,* and the *Enterobacter cloacae* complex (Table 1). The sequence types (STs) of *K. pneumoniae* were determined by analyzing seven housekeeping genes, including *rpoB*, *gapA*, *mdh*, *pgi*, *phoE*, *infB*, and *tonB* (Diancourt et al., 2005). The *E. coli* MLST scheme uses internal fragments of seven housekeeping genes: *adk*, *fumC*, *gyrB*, *icd*, *mdh*, *purA*, and *recA* (Wirth et al., 2006). The *dnaA*, *fusA*, *gyrB*, *leuS*, *pyrG*, *rplB*, and *rpoB* genes were amplified and analyzed for clonal lineages of *E. cloacae* (Miyoshi-Akiyama et al., 2013). STs were assigned using the following MLST databases: https://bigsdb.pasteur.fr/klebsiella, https://mlst.warwick.ac.uk/mlst/dbs/Ecoli, and https://pubmlst.org/ecloacae for *E. cloacae*.

**Table 1:** Bacterial species, genotype, and source of carbapenemase-producing *Enterobacterales* isolates for multi-locus sequence typing analysis.

Species	Genotype	Source	
		Stream (N^*^)	Patient (*N*)
*Klebsiella pneumoniae*	*bla*_KPC-2_	21	154
	*bla*_KPC-3_	2	2
	*bla*_NDM-1_	1	15
	*bla*_NDM-5_	11	3
*Escherichia coli*	*bla*_NDM-1_	1	3
	*bla*_NDM-5_	14	5
*Enterobacter cloacae* complex	*bla*_KPC-2_	3	5
Total		53	187

* N: Number of isolates.

## Results

### Prevalence and Antimicrobial Resistance of CRE Strains

A total of 133 CRE strains were isolated from 21 (22.3%) streams in Korea. *K. pneumoniae* was the most common CRE (*n* = 61; 45.9%), followed by the *E. cloacae* complex (*n* = 39; 29.3%), *E. coli* (*n* = 18; 13.5%), *Raoultella ornithinolytica* (*n* = 7; 5.3%), and *Citrobacter freundii* (*n* = 3; 2.3%; Table 2).

**Table 2:** Distribution of carbapenem-resistant *Enterobacterales* isolates from streams.

Species	No. (%) of isolates
*Klebsiella pneumoniae*	61 (45.9)
*Enterobacter cloacae* complex	39 (29.3)
*Escherichia coli*	18 (13.5)
*Raoultella ornithinolytica*	7 (5.3)
*Citrobacter freundii*	3 (2.3)
*Klebsiella oxytoca*	2 (1.5)
*Kluyvera cryocrescens*	1 (0.8)
*Lelliottia amnigena*	1 (0.8)
*Serratia marcescens*	1 (0.8)

**Figure 1:**
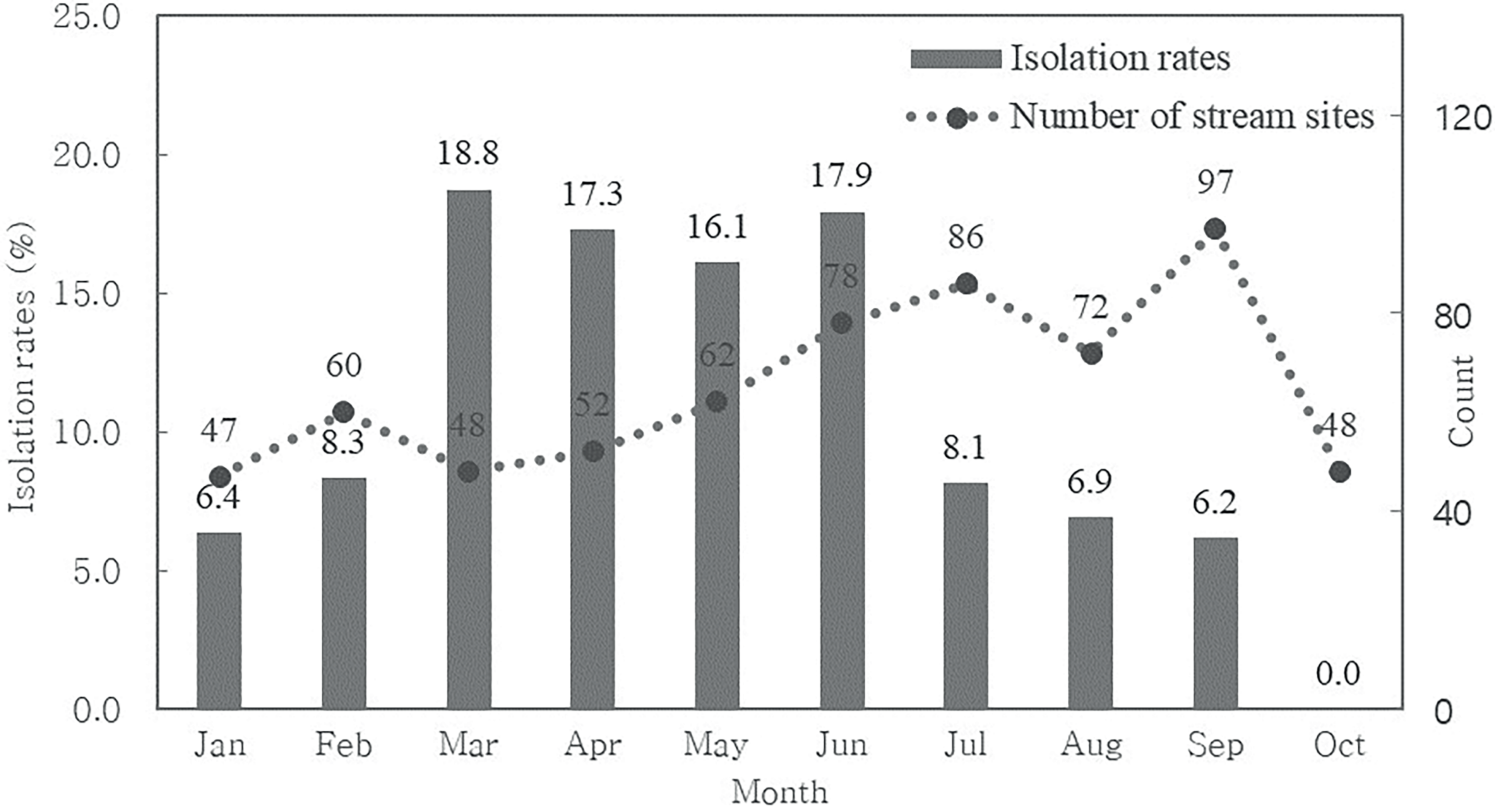
Monthly carbapenem-resistant *Enterobacterales* isolation rates for stream sites.

We calculated the monthly CRE isolation rate to determine seasonal variation (Figure 1). We found that CRE was continuously isolated from streams throughout the year, although we did not collect water from streams between November and December.

**Figure 2:**
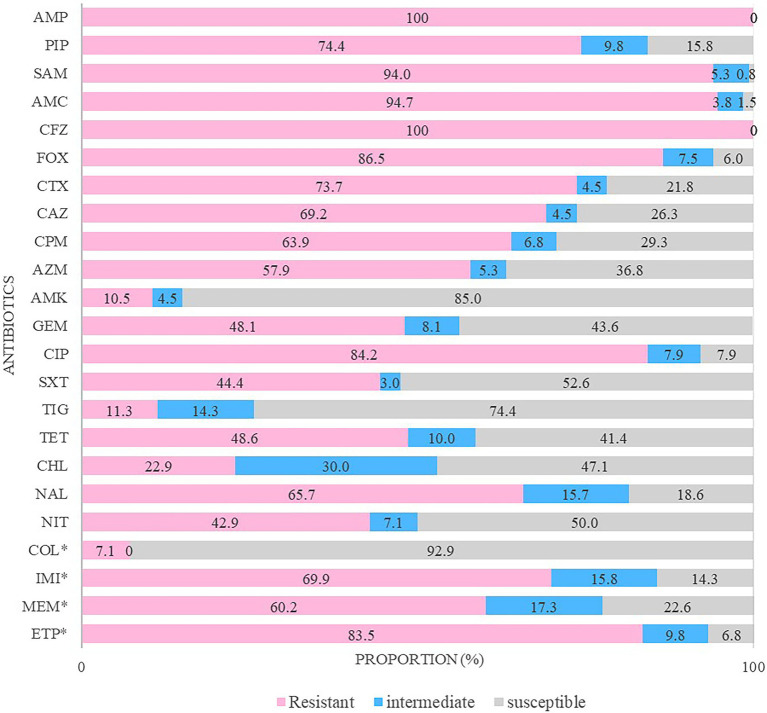
Antimicrobial resistance patterns of carbapenem-resistant Enterobacterales isolates. The antibiotics tested in this study were ampicillin (AMP), piperacillin (PIP), ampicillin/sulbactam (SAM), amoxicillin/clavulanic acid (AMC), cefazolin (CFZ), cefoxitin (FOX), cefotaxime (CTX), ceftazidime (CAZ), cefepime (CPM), aztreonam (AZM), amikacin (AMK), gentamicin (GEM), ciprofloxacin (CIP), trimethoprim/sulfamethoxazole (SXT), tigecycline (TIG), tetracycline (TET), chloramphenicol (CHL), nalidixic acid (NAL), nitrofurantoin (NIT), colistin (COL), imipenem (IMI), meropenem (MEM), and ertapenem (ETP). ^*^ They were determined by minimum inhibitory concentration (MIC).

The resistance to ertapenem was higher (83.5%) than that to imipenem (69.9%) and meropenem (60.2%; Figure 2). Almost all CRE isolates were highly resistant to various antimicrobial agents, including ampicillin–sulbactam (94.0%), amoxicillin/clavulanic acid (94.7%), ciprofloxacin (89.5%), and cefotaxime (73.7%). Moreover, 100% of the CRE isolates were resistant to ampicillin and cefazolin, whereas 89.5% showed high susceptibility to amikacin.

### Molecular Characterization of Carbapenemase Genes

Among the 133 CRE isolates, carbapenemase genes were found in 90 (67.7%). All isolates of *R. ornithinolytica, C. freundii, Klebsiella oxytoca,* and *Kluyvera cryocrescens* were CPE. In other strains, carbapenemase genes were detected in 51 of 61 *K. pneumoniae* isolates, 17 of 18 *E. coli* isolates, and 9 of 39 *E. cloacae* isolates.

The most common genotypes were KPC-2 (*n* = 34; 37.8%), New Delhi metallo-β-lactamase (NDM)-5 (*n* = 29; 32.2%), Guiana extended-spectrum (GES)-5 (*n* = 9; 10.0%), NDM-1 (*n* = 7; 7.8%), oxacillinase (OXA)-48 (*n* = 7; 7.8%), KPC-3 (*n* = 3; 3.3%), and GES-6 (*n* = 1; 1.1%), respectively (Table 3). Of the 90 CPE isolates, there were 29 KPC-2-producing *K. pneumoniae,* 15 NDM-5-producing *E. coli*, 11 NDM-5-producing *K. pneumoniae*, and 7 OXA-48-producing *K. pneumoniae* isolates.

**Table 3:** Distribution of carbapenemase genotypes in carbapenemase-producing *Enterobacterales* isolates from streams.

Species	Number (%) of isolates							
	Total	*bla*_KPC-2_	*bla*_KPC-3_	*bla*_NDM-1_	*bla*_NDM-5_	*bla*_GES-5_	*bla*_GES-6_	*bla*_OXA-48_
*K. pneumoniae*	51	29 (56.9)	2 (3.9)	1 (2.0)	11 (21.6)	0	1 (2.0)	7 (13.7)
*E. coli*	17	0	0	1 (5.9)	15 (88.2)	1 (5.9)	0	0
*R. ornithinolytica*	7	0	1 (14.3)	0	2 (28.6)	4 (57.1)	0	0
*E. cloacae* complex	5	3 (60.0)	0	0	1 (20.0)	1 (20.0)	0	0
*E. kobei*	3	1 (33.3)	0	0	0	2 (66.7)	0	0
*C. freundii*	3	0	0	3 (100)	0	0	0	0
*K. oxytoca*	2	0	0	2 (100)	0	0	0	0
*E. hormaechei*	1	0	0	0	0	1 (100)	0	0
*K. cryocrescens*	1	1 (100)	0	0	0	0	0	0
Total	90	34 (37.8)	3 (3.3)	7 (7.8)	29 (32.2)	9 (10.0)	1 (1.1)	7 (7.8)

### Genetic Relatedness

A total of 53 CPE isolates isolated from the stream environment were analyzed using MLST for KPC-2, KPC-3, NDM-1, and NDM-5-producing *K. pneumoniae*; NDM-1 and NDM-5-producing *E. coli*; and KPC-2-producing *E. cloacae* complex isolates (Table 4). ST307 (*n* = 16) was the most common ST in KPC-2-producing *K. pneumoniae* isolates, followed by ST11 (*n* = 2), ST1255 (*n* = 1), ST2012 (*n* = 1), and a new ST (*n* = 1). Four STs and one new ST were found in 11 NDM-5-producing *K. pneumoniae*, and ST2830 (*n* = 6) was the most common ST. Furthermore, 14 *E. coli* strains producing NDM-5 were classified as ST744 (*n* = 5), ST405 (*n* = 3), ST359 (*n* = 2), and ST2659, ST205, ST3058, and ST617 (*n* = 1 each). ST484, ST520, and ST910 (*n* = 1 each) were found in the *E. cloacae* complex isolates that produce KPC-2.

**Table 4:** Multi-locus sequence typing of carbapenemase-producing *Klebsiella pneumoniae*, *Escherichia coli*, and *Enterobacter cloacae* strains from streams and patients.

Species	Genotypes	MLST	
		Stream (N^*^)	Patient (*N*)
*K. pneumoniae*	*bla*_KPC-2_	ST307(16), ST11(2), ST1255(1), ST2012(1), New(1)	ST307(80), ST11(42), ST48(23), ST789(3), ST15(2), ST273(1), ST392(1), New(2)
	*bla*_KPC-3_	ST461(1), ST1699(1)	ST307(1), ST668(1)
	*bla*_NDM-1_	ST202(1)	ST307(9), ST35(1), ST147(1), ST1488(4)
	*bla*_NDM-5_	ST2830(6), ST515(2), ST22(1), ST1994(1), New(1)	ST307(1), ST2830(1), ST2294(1)
*E. coli*	*bla*_NDM-1_	ST156(1)	ST297(1), ST131(1), ST38(1)
	*bla*_NDM-5_	ST744(5), ST405(3), ST359(2), ST2659(1), ST205(1), ST3058(1), ST617(1)	ST410(2), ST405(1), ST2659(1), ST48(1)
*E. cloacae*	*bla*_KPC-2_	ST484(1), ST520(1), ST910(1)	ST78(4), ST484(1)

* N: Number of isolates.

We included 187 clinical isolates from patients in the MLST analysis. ST307 (*n* = 80) was the most common ST in 154 KPC-2-producing *K. pneumoniae* strains, followed by ST11 (*n* = 42), ST48 (*n* = 23), ST789 (*n* = 3), ST15 (*n =* 2), ST273 (*n* = 1), ST392 (*n* = 1), and a new ST (*n* = 2), respectively. ST307, ST2830, and ST2294 (*n* = 1 each) were found in NDM-5-producing *K. pneumoniae*. In addition, 5 *E. coli* strains producing NDM-5 were classified as ST410 (*n* = 2), ST405, ST2659, and ST48 (*n* = 1 each). Furthermore, 5 *E. cloacae* complex isolates producing KPC-2 were classified as ST78 (*n* = 4) and ST484 (*n* = 1).

The major STs (ST307, ST11, and ST2830) of the KPC-2/NDM-5-producing *K. pneumoniae* isolates from the streams were consistent with those from the same strains isolated from patients. NDM-5-positive *E. coli* with ST405/ST2659 and KPC-2-positive *E. cloacae* complex with ST485 were detected simultaneously in isolates from the streams and patients.

## Discussion

Antimicrobial resistance is a major global health concern. The ecosystem surrounding humans is considered an important route of resistance transmission based on the One Health concept (Robinson et al., 2016). We investigated the presence of CRE and CPE in streams and studied the epidemiological relationship between CPE isolates from streams and those from patients.

We detected 133 CRE isolates from 21 (22.3%) of 94 streams located in Korea between July 2017 and August 2019. Among these, 88.7% of the strains were isolated from urban streams that cross cities. Urban streams flowing through densely populated cities can be a source of antimicrobial resistance owing to fecal pollution (Reynolds et al., 2020). Of note, a specific stream which was located in the most populous administrative district showed the highest prevalence of CRE (14.0%; data not shown). In this study, 67.7% of the CRE isolates were CPE. *K. pneumoniae* (56.7%) and *E. coli* (18.9%) were the two most common CPE isolated from streams. These results were similar to a previous report on the prevalence and characteristics of CPE in Korea, although the third most common CPE (*R. ornithinolytica*) isolated from the stream samples is uncommon in patients (Kim et al., 2020). Therefore, we concluded that there is a close association between the presence of CPE in streams and humans.

Recently, the global spread of KPC, NDM, and OXA-48 has become a serious challenge in many countries (Grundmann et al., 2017; Yoon et al., 2018b). CPE can transmit resistance genes to other bacteria *via* horizontal transfer. Therefore, streams containing these CPE strains can serve as reservoirs for resistance genes. The detection of CPE genes, such as *bla_NDM-1_* in seepage and tap water, *bla_NDM-5_*, and *bla_NDM-7_* in Indian rivers, and *bla_OXA-48_* in wastewater, has been reported (Walsh et al., 2011; Akiba et al., 2016; Muller et al., 2018). In our study, we detected several CPE genes, including *bla_KPC-2_*, *bla_KPC-3_*, *bla_NDM-1_*, *bla_NDM-5_*, *bla_GES-5_*, *bla_GES-6_*, and *bla_OXA-48_*, from stream samples, and *bla_KPC-2_* and *bla_NDM-5_* were the most prevalent. The prevalence of CPE genes in the streams was very similar to that in patients, although *bla_NDM-5_* was more prevalent in streams than in patients (Kim et al., 2020).

To determine the genetic relatedness between the stream and clinical isolates, MLST analysis was performed for *K. pneumoniae, E. coli,* and *E. cloacae* isolates. ST258 is the most common ST in clinical isolates, especially in United States (Kitchel et al., 2009). However, ST11, a single-locus variant of ST258, is the predominant clone in China (Qi et al., 2011). In this study, ST307 and ST11 were the common clone types in KPC-2-producing *K. pneumoniae* isolated from streams, and these STs were also prevalent in patient isolates. Similarly, Yoon et al. reported that KPC-producing *K. pneumoniae* harboring ST307 and ST11 were most common in Korea (Yoon et al., 2018a). Although several STs were detected in stream samples but not found in the patient samples, these results indicate that CPE, especially KPC-2-producing *K. pneumoniae*, from stream environments, are closely related to those from patients.

In summary, we detected 133 CRE from 21 streams, including 90 CPE isolates which produced KPC-2, KPC-3, NDM-1, NDM-5, GES-5, GES-6, and OXA-48. The prevalence of CPE genes in the streams was similar to that in the patients. Notably, KPC-2-producing *K. pneumoniae* infections were the most common. ST307 and ST11 were the most common clone types among these isolates. The origin of CPE separated from the stream could not be clearly concluded. However, considering that the CPE gene of Enterobacterales from clinical samples is similar to the that from the stream, it was estimated that CPE from the clinical sample is closely related to CPE from the stream. We believe that systematic and continuous monitoring of stream environments is required to control the spread of antimicrobial resistance.

## Data Availability Statement

The original contributions presented in the study are included in the article/supplementary material, further inquiries can be directed to the corresponding author.

## Author Contributions

G-HS and SiK designed the study, analyzed the data, and wrote the original draft. EP, SH, J-DK, GK, and E-YK performed the sample collection and experiments. JJ and SuK provided the clinical strains and reviewed the manuscript. JS also assisted with the study design, writing, review, and editing. All authors contributed to the article and approved the submitted version.

## Funding

This research was supported by a fund (2017ER540202) by Research of Korea Centers for Disease Control and Prevention and a grant of the Korea Health Technology R&D Project through the Korea Health Industry Development Institute (KHIDI) and funded by the Ministry of Health & Welfare, Republic of Korea (grant number: HR21C1003).

## Conflict of Interest

The authors declare that the research was conducted in the absence of any commercial or financial relationships that could be construed as a potential conflict of interest.

## Publisher’s Note

All claims expressed in this article are solely those of the authors and do not necessarily represent those of their affiliated organizations, or those of the publisher, the editors and the reviewers. Any product that may be evaluated in this article, or claim that may be made by its manufacturer, is not guaranteed or endorsed by the publisher.
